# Massively parallel pyrosequencing highlights minority variants in the HIV-1 env quasispecies deriving from lymphomonocyte sub-populations

**DOI:** 10.1186/1742-4690-6-15

**Published:** 2009-02-12

**Authors:** Gabriella Rozera, Isabella Abbate, Alessandro Bruselles, Crhysoula Vlassi, Gianpiero D'Offizi, Pasquale Narciso, Giovanni Chillemi, Mattia Prosperi, Giuseppe Ippolito, Maria R Capobianchi

**Affiliations:** 1 Laboratory of Virology, INMI L. Spallanzani, Rome, Italy; 2 Clinical Department, INMI L. Spallanzani, Rome, Italy; 3 Consorzio Interuniversitario per le Applicazioni di Supercalcolo per l'Università e la Ricerca (CASPUR), Rome, Italy; 4 Scientific Direction, INMI L. Spallanzani, Rome, Italy

## Abstract

**Background:**

Virus-associated cell membrane proteins acquired by HIV-1 during budding may give information on the cellular source of circulating virions. In the present study, by applying immunosorting of the virus and of the cells with antibodies targeting monocyte (CD36) and lymphocyte (CD26) markers, it was possible to directly compare HIV-1 quasispecies archived in circulating monocytes and T lymphocytes with that present in plasma virions originated from the same cell types. Five chronically HIV-1 infected patients who underwent therapy interruption after prolonged HAART were enrolled in the study. The analysis was performed by the powerful technology of ultra-deep pyrosequencing after PCR amplification of part of the *env *gene, coding for the viral glycoprotein (gp) 120, encompassing the tropism-related V3 loop region. V3 amino acid sequences were used to establish heterogeneity parameters, to build phylogenetic trees and to predict co-receptor usage.

**Results:**

The heterogeneity of proviral and viral genomes derived from monocytes was higher than that of T-lymphocyte origin. Both monocytes and T lymphocytes might contribute to virus rebounding in the circulation after therapy interruptions, but other virus sources might also be involved. In addition, both proviral and circulating viral sequences from monocytes and T lymphocytes were predictive of a predominant R5 coreceptor usage. However, minor variants, segregating from the most frequent quasispecies variants, were present. In particular, in proviral genomes harboured by monocytes, minority variant clusters with a predicted X4 phenotype were found.

**Conclusion:**

This study provided the first direct comparison between the HIV-1 quasispecies archived as provirus in circulating monocytes and T lymphocytes with that of plasma virions replicating in the same cell types. Ultra-deep pyrosequencing generated data with some order of magnitude higher than any previously obtained with conventional approaches. Next generation sequencing allowed the analysis of previously inaccessible aspects of HIV-1 quasispecies, such as co-receptor usage of minority variants present in archived proviral sequences and in actually replicating virions, which may have clinical and therapeutic relevance.

## Background

The error prone nature of HIV-1 reverse transcriptase, combined with the high replicative activity of the virus, results, in each infected individual, in the formation of many genetically related viral variants referred to as quasispecies, in which most viral sequences differ from all others. This variability is the substrate for the selective pressure exerted by drugs or by the immune system, leading to the continuous evolution of HIV-1 in the infected host [1,2]. The most variable part of the HIV-1 genome is the region coding for the V3 loop of HIV-1 surface glycoprotein (gp120) that is involved in the coreceptor binding [3]. Shortly after primary infection, viral heterogeneity is relatively low and progressively increases in the absence of treatment [4,5]. During the natural history of the infection, compartmentalized viral replication in different cell types may contribute to virion diversity, and ultimately may determine the segregation of viral clusters in different body sites [6-9]. While recent reports show that in patients treated with antiviral drugs HIV-1 quasispecies present in monocytes may evolve in clusters segregated from viral quasispecies harboured by lymphocytes [10,11], most HIV-1 compartmentalization studies have focused mainly on proviral DNAs in lymphomonocyte populations [10-16].

However, HIV-1 proviral DNA represents an archive of viral variants, including those acquired in the past, that may not necessarily reflect the viral population replicating at every given time, which makes the evaluation of how the different cell sources impact the circulating HIV-1 quasispecies rather difficult.

Cell-derived antigens acquired during the budding process serve as markers of the virus cellular origin [17-21]. Consequently, using cell type-specific antibodies when studying plasma virions may aid in identifying viral populations originating *in vivo *from different cellular sources [19-22]. In the present study, we analyzed five patients who experienced therapy interruptions after prolonged periods of highly active antiretroviral therapy (HAART). Stringent inclusion criteria are detailed in the Materials and Methods section. Considering that, as recently shown, both activated immature monocyte/macrophage (CD36 positive) and CD4 T cell (CD26 positive) compartments contribute to viral load [22], proviral V3 quasispecies harboured by these cells at therapy interruption (T0) were compared to quasispecies present in circulating viral RNA genomes one month later (T1). Monocyte- and lymphocyte-enriched cell sources were obtained by immunosorting with anti-CD36 and anti-CD26 monoclonal antibodies; the same antibodies were used to sort circulating virions originated from the same cell lineages as previously described [19].

To study the V3 quasispecies, an innovative and powerful technology was used: the ultra-deep pyrosequencing, performed with the 454 Life Sciences platform (GS-FLX, distributed by Roche). By this approach it is possible to analyze simultaneously thousands of clonally amplified PCR amplicons, increasing the probability of identifying minority variants, as already shown in [23-25] for rare HIV drug resistance mutations.

After sequencing, heterogeneity parameters were calculated for both proviral and circulating virion amino acid sequences. Phylogenetic analysis was performed to identify the genetic relationship between viral genomes from different sources. Co-receptor usage was deduced from the V3 region sequence of each variant.

## Materials and methods

### Patients

The enrolled subjects were 5 chronically HIV-1 infected patients who underwent therapy interruption after prolonged treatment with effective HAART (overall extent ≥ 5 years, with at least 2 consecutive years before enrollment). Therapeutic regimens included combinations of two NRTIs and either an NNRTI or a ritonavir-boosted PI. Pt. 2 underwent a therapy interruption cycle 3 years before the present study.

The eligibility criteria to interrupt HAART were: CD4^+^>350/microliter; nadir CD4^+^>100/microliter; absence of virological failure during the last HAART course (≥ 2 years); CDC classification: A or B. The project was approved by the Institutional Ethics Committee, and the patients agreed to participate by signing an informed consent. Demographic, clinical and virological data are reported in Table 1. Plasma viremia was determined using the Versant HIV RNA test, version 3.0 (Siemens Medical Solutions).

**Table 1:** Demographic, clinical and virological features of the study patients

**Patient**	**Age** **(yrs)**	Gender*	**Time of** **Infection** **(yrs)**	**Total time on** **HAART** **(yrs)**	**NADIR CD4** **(cells/microliter**	**CD4** T0** (cells/microliter)	**HIV-RNA** T0** (Log_10_**cp/ml)**	**CD4** T1*** (cells/microliter)	**HIV-RNA** T1*** (Log_10_**cp/ml)**
Pt.1	48	F	18	6	312	569	<1.7	598	3,30

Pt.2	37	M	11	10	438	1093	<1.7	1162	1,92

Pt.3	52	M	8	7	336	699	<1.7	520	> 5,70

Pt.4	41	M	10	9	246	697	<1.7	428	5,25

Pt.5	50	F	17	9	223	792	<1.7	511	5,60

* M, male; F, female.

** T0: at the time of HAART interruption

*** T1: one month after HAART interruption

All patients were infected with HIV-1 subtype B, as determined by sequence analysis of *env *region, according to the Los Alamos genotyping algorithm.

### Immunosorting of lymphomonocyte subpopulations and of HIV-1 virions

Monocytes and T lymphocyte cells were purified by immunomagnetic sorting (Miltenyi Biotec, Bologna, Italy) from freshly isolated peripheral blood mononuclear cells (PBMC) by positive selection using anti-CD36 (clone CLB-703, Monosan) antibody for monocytes and anti CD26 (clone M-A261, Pharmingen) antibody for T-cells.

HIV-1 virions from patients' plasma, originating from either monocytes or T lymphocytes, were sorted by immobilized antibody capture (IAC), using the same antibodies used for PBMC sorting (anti CD36 and anti CD26 antibodies) as described elsewhere [21]. To rule out the possibility that soluble molecules present in the fluids could inhibit virus binding to specific MAbs, IAC was performed on HIV-1 purified according to published procedures [19] by applying 50 μl of purified virion preparations, diluted as appropriate to contain 50,000–100,000 RNA copies/ml, on 96 wells PRO-BIND Assay Plates (Becton Dickinson) coated with the specific Mabs. As IAC yield for each antibody ranged between 5 and 10% of input virus, to obtain sufficient amounts of virions to undergo ultra-deep pyrosequencing, HIV-1 captured by 10 different wells coated with either anti-CD36 and or anti-CD26 were pooled.

To validate the method used to sort the viral quasispecies originating from the two cellular sources, two HIV-1 strains, genetically distinct and with different co-receptor usage (i.e. the reference R5 strain HIV-1 BaL and a clinical X4 isolate) were grown on monocytes derived macrophages (MDM) and on PHA activated CD4+ T lymphocytes, respectively. The R5 and the X4 viral preparations were mixed at a ratio of 1:9 and applied to 2 sets of immuno-capture wells, coated with anti-CD36 and anti-CD26 antibodies, respectively. Then the immuno-captured virions were analyzed by ultra-deep pyrosequencing. By this approach, only 0.51% of virions captured by anti-CD36 actually represented virions with a V3 sequence matching that of the X4 strain grown on CD4+ T lymphocytes; in parallel, only 0.12% of virions captured by anti-CD26 segregated with the R5 strain grown on MDM. These results indicate that the cell lineage-specific antibodies in fact captured the virions originating from the corresponding cellular subpopulation, with high specificity (see Additional file 1).

To further establish the specificity of the virion immunocapture method, binding to the control antibody anti-CD19 was evaluated. The proportion of virions captured by anti-CD19 was <0.001% for R5 strain grown on MDM and 0.195% for the X4 strain grown on CD4+ T lymphocytes. The proportion of plasma virions from the study patients captured by the control antibody was consistently low (in the range of 0.3–0.7%).

### Nucleic acid extraction, RT and PCR conditions

Total DNA extraction from CD36 and CD26 cells was performed by using a DNA blood kit (Qiagen, Hilden, Germany). HIV-1 RNA from immunocaptured virions underwent extraction using the QIAamp Viral RNA kit (Qiagen). Retrotranscription was performed by random hexamer extension with *M-MuLV *Reverse Transcriptase (Roche) for 1 h at 42°C followed by 15 min at 65°C.

For V3 region amplifications, two rounds of 35 cycles (95°C for 2 min, 95° for 30 sec, annealing at 60°C for 30 sec, extension at 72°C for 45 sec and final elongation at 72°C for 5 min.) were carried out using a proof-reading enzyme (Fast Start High fidelity enzyme, Roche): outer sense primer *ATGGGATCAAAGCCTAAAGCCATGTG *(position 6556–6581 in HXB2), outer antisense primer *AGTGCTTCCTGCTGCTCCCAAGAACCCAAG *(position 7822–7792 in HXB2), inner sense primer ***GCCTCCCTCGCGCCATCAG ****TGGCAGTCTAGCAGAAGAAG *(position 7010 to 7029 in HXB2) and inner antisense primer ***GCCTTGCCAGCCCGCTCAG****CTGGGTCCCCTCCTGAGG *(position 7332 to 7315 in HXB2). The inner primers included 5' extensions (underlined) which provided binding sites for pyrosequencing (see below).

To maximize the number of templates undergoing ultra-deep pyrosequencing, we pooled a number of 2 to 10 different PCR reactions for each sample type.

In addition, in order to avoid possible cross-contamination of the amplicons, each sample was handled in separate time frames, and accurate decontamination was performed after each manipulation of the nucleic acids.

### Ultra-deep pyrosequencing

Ultra-deep pyrosequencing was carried out with the 454 Life Sciences platform (GS-FLX, Roche Applied Science). PCR products were clonally amplified on capture beads in water-in-oil emulsion micro-reactors, and pyrosequencing was performed by using one of 16 lanes of a 70 × 75 mm PicoTiterPlate for each sample, following the standard approach for PCR amplicons sequencing. For each sample an SFF file was obtained, from which nucleotide sequence data were extracted.

### UDPS error rate estimation and correction algorithm

To measure the accuracy of the ultra-deep pyrosequencing, a plasmid clone containing the region of interest was sequenced in parallel by ultra-deep pyrosequencing and by the Sanger method. The plasmid clone was obtained from a patient's sample by inserting a PCR amplicon spanning nucleotide positions 6,989 to 7,667 (reference strain HXB2) into a pCR4-TOPO vector (Invitrogen Corp.). Sanger sequencing of the clone was performed on ABI Prism 310, using the BigDye Terminator cycle sequencing kit, following the manufacturer's instructions (Applied Biosystems Warrington, UK). Any differences between the two methods were considered to be a GS-FLX sequencing error. Because it has been previously reported that the pyrosequencing error rate is higher in regions with nucleotide repeats of three or more identical bases, defined as homopolymeric [26], we determined the error rates separately in homopolymeric and in non-homopolymeric regions. The error rate in homopolymeric regions (3 to 5 identical nucleotides) was 0.0097 ± 0.0056 (mean ± SEM), whereas in non-homopolymeric regions it was 0.0024 ± 0.0009; the overall error rate was 0.0043 ± 0.0016.

To be noted, the plasmid used for the evaluation contained a highly polymeric region of 6 adjacent adenines (A), that was read as an incomplete extension of 5 A-homopolymer (Additional file 2). However, this particular pattern was present only in CD36-virus from one patient, where it represented 21% of the total clones from this source. Of these, only 3% were read well, whereas the remaining 18% showed a 5 A-homopolymer. The latter situation introduced a stop codon in the sequences, determining their elimination during the subsequent correction process of the sequences. Therefore the error appeared to have no consequence on the overall results.

Based on these considerations, and with the aim of considering only the sequences leading to functional products, we adopted the following correction algorithm. Nucleotide sequences from each sample were divided into two separate files, one for the forward reads and one for the reverse ones. These sequences were then translated into amino acids with EMBOSS [27] using all possible frames; only those translated with the right open reading frame (ORF) were retained.

Multiple alignments of the amino acid sequence files were then constructed using the software MUSCLE [28] (default options) and trimmed at the 5' and 3' termini to include only a region potentially covered by both forward and reverse reads. The *env *region resulting from this trimming consisted of about 66 amino acids and encompassed the V3 loop (-16 before and +15 after V3 loop).

To reduce the error rate, sequences containing ambiguous bases (Ns) were also discarded; this reduced the error rate due to the possible presence of reads coming from multi-templated beads [29]. Then, for each sample we compared the forward sequence datasets with the reverse ones and clustered them, reporting only identical matches between the two.

The application of the correction algorithm led to a reduction in the number of sequences for each sample type that underwent quasispecies analysis (see Additional file 3); the unique variants for each sample type obtained after de-replication were used for the construction of the phylogenetic trees.

All the sequences obtained from the patients have been compared to the sequences of reference HIV strains present in the laboratory to rule out possible contamination with these amplicons.

### Heterogeneity parameters calculation and construction of phylogenetic trees

After the application of the above mentioned sequence correction algorithm, the resulting amino acid sequences were used for quasispecies analysis. Although nucleotide sequences provide more information to the end of heterogeneity and phylogenetic analyses, the choice to use amino acid sequences was forced by the limitation of existing bioinformatic tools, designed for medium-small scale size of data sets. However, we were able to perform a limited phylogenetic analysis based on nucleotide sequences from the patient displaying the smallest number of unique variants (i.e. pt 4), obtaining a tree shape substantially equivalent to that based on amino acid sequences (not shown). To assess diversity, the mean genetic distance of amino acid sequences was calculated by PROTDIST using Jones-Taylor-Thornton matrix and with an in-house written code. Quasispecies complexity was calculated using normalized Shannon (S) entropy [21]. The neighbor-joining method was used to construct individual phylogenetic trees for each patient. Bootstrap analysis of 1,000 replicates was used to place approximate confidential limits on individual branches. All the algorithms for quasispecies analysis were included in the MEGA package Version 4.0.1. Although the individual patients' trees are built using only unique variants in each sample type (CD36-provirus, CD26-provirus, CD36-virus, CD26-virus), the relative abundance of sequences included in the clusters and their mean PSSM score were referred to all the variants in each sample type.

### Prediction of coreceptor usage by position specific score matrices (PSSM)

PSSM analysis for HIV-1 subtype B was applied to the V3 amino acid sequences obtained by ultra-deep pyrosequencing to obtain a score for co-receptor usage prediction as described elsewhere [30]. In general, the higher the score, the higher the probability that the given V3 sequence uses CXCR4 co-receptor. The 95th and 5th percentiles for X4 and R5 tropism are > -2.88 and < -6.96, respectively. As reference, the score of the R5 strain HIV-1 BaL is -12.96, while the score of the X4 strain HIV-1 HXB2 is +3.47.

## Results

### V3 loop heterogeneity in HIV from monocytes and T lymphocytes

The characteristics of the 5 study patients are reported in Table 1. Four types of samples were analyzed: 1) proviral DNA from monocytes (CD36-provirus); 2) proviral DNA from T lymphocytes (CD26-provirus); 3) viral RNA from anti CD36-captured virions (CD36-virus); 4) viral RNA from anti CD26-captured virions (CD26-virus). For 2 patients (Pt. 1 and Pt. 2) the analysis was restricted to provirus, since the rebounding HIV-1 viremia was too low to perform immuno-capture and subsequent quasispecies analysis.

The GS-FLX sequencing platform generated an average of 13,456 reads per sample, determining a median coverage of 12,609 reads per nt. A correction algorithm, substantially based on the translation in amino acid sequences and on the retention of only coding sequences, was applied. The number of filtered amino acid sequences used for quasispecies analysis is reported in Additional file 3. The application of this algorithm restricted the analysis only to sequences with a phenotypic significance and at the same time reduced the computational burden, allowing us to use conventional softwares for phylogenetic analysis.

The number of unique variants, diversity and complexity of HIV-1 V3 loop for CD36-provirus, CD26-provirus, CD36-virus and CD26-virus are shown in Table 2. Diversity was significantly correlated with complexity (r = 0.72, p = 0.0017, in Pearson correlation test). All these parameters in the monocyte compartment (including proviral and viral HIV-1) were generally higher than those found in T-lymphocytes. In fact, with the exception of one patient (Pt.1), in all patients unique proviral sequences deriving from CD36 cells were more numerous than those harboured by CD26 cells; similarly, with the exception of one patient (Pt 4), the diversity was higher in CD36 provirus as compared to CD26 provirus. Concerning circulating virions, the situation was more variable. On the whole, mean diversity of proviral+viral sequences of CD36-derived HIV-1 was significantly higher than CD26-derived HIV-1 (0.060 ± 0.015 vs 0.022 ± 0.006, p = 0.027 in Student's t test).

**Table 2:** Amino acid heterogeneity of V3 region of HIV-1 proviral quasispecies harboured by monocytes (CD36) and T lymphocytes (CD26) and of plasma virions replicating in the two cell types after HAART interruption.

**Patient**	**Sample type**		N. of unique variants*	Diversity**	Complexity***
Pt.1	Provirus	CD36	161	0.0795 ± 0.0005	0.3798
		
		CD26	226	0.0197 ± 0.0001	0.2119

Pt.2	Provirus	CD36	231	0.1446 ± 0.0005	0.3620
		
		CD26	59	0.0031 ± 0.0001	0.0919

Pt.3	Provirus	CD36	46	0.0508 ± 0.0003	0.3042
		
		CD26	3	0.0084 ± 0.0002	0.0738
	
	Virus	CD36	26	0.0428 ± 0.0010	0.2979
		CD26	158	0.0298 ± 0.0003	0.3293

Pt.4	Provirus	CD36	76	0.0226 ± 0.0007	0.1127
		
		CD26	14	0.0457 ± 0.0003	0.1755
	
	Virus	CD36	15	0.0113 ± 0.0002	0.1138
		
		CD26	41	0.0208 ± 0.0002	0.1222

Pt.5	Provirus	CD36	157	0.0585 ± 0.0003	0.2756
		
		CD26	120	0.0411 ± 0.0004	0.4233
	
	Virus	CD36	305	0.0728 ± 0.0005	0.3664
		
		CD26	38	0.0042 ± 0.0001	0.0870

Sequences obtained by ultra-deep pyrosequencing were filtered through the correction algorithm and amino acid sequences obtained were analyzed to establish diversity and complexity, as described in the Materials and Methods section.

* Number of unique sequences obtained after dereplication

** Diversity: p distance ± SEM

*** Complexity: normalized Shannon entropy

### Individual phylogenetic analysis of HIV-1 env region from monocytes and T lymphocytes

**Figure 1:**
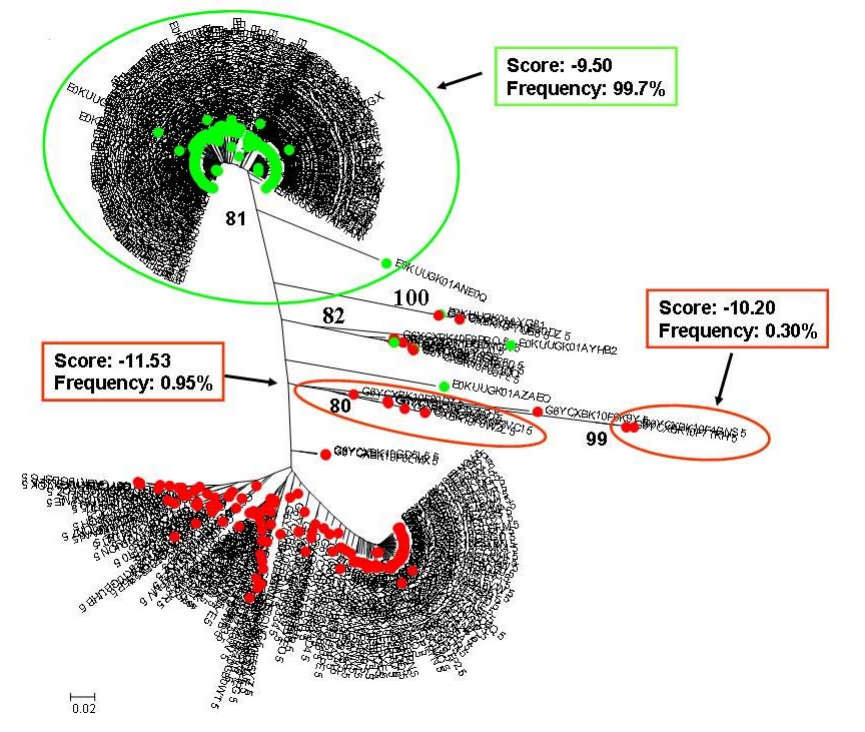
**Individual phylogenetic tree of HIV-1 V3 amino acid sequences from Pt.1**. Proviral quasispecies harboured by monocytes (red circle = CD36-provirus) and T lymphocytes (green circle = CD26-provirus) of Pt.1 were analyzed after long term suppression of viremia. Nucleotide sequences obtained by ultra-deep pyrosequencing were translated in amino acid sequences and filtered through the correction algorithm described in the Material and Methods section. Unique sequences for each sample type were used to build phylogenetic trees with the neighbour-joining method. Bootstrap values ≥ 80% are shown. The cellular sources of proviral and viral sequences are indicated by coloured bullets. Sample type-specific cluster with bootstrap values ≥ 80% are encircled with the corresponding colours. PSSM score was calculated for each of these clusters, and the corresponding values are included in the insert, together with their relative abundance in the corresponding sample type. Bars indicate p distance scale.

**Figure 2:**
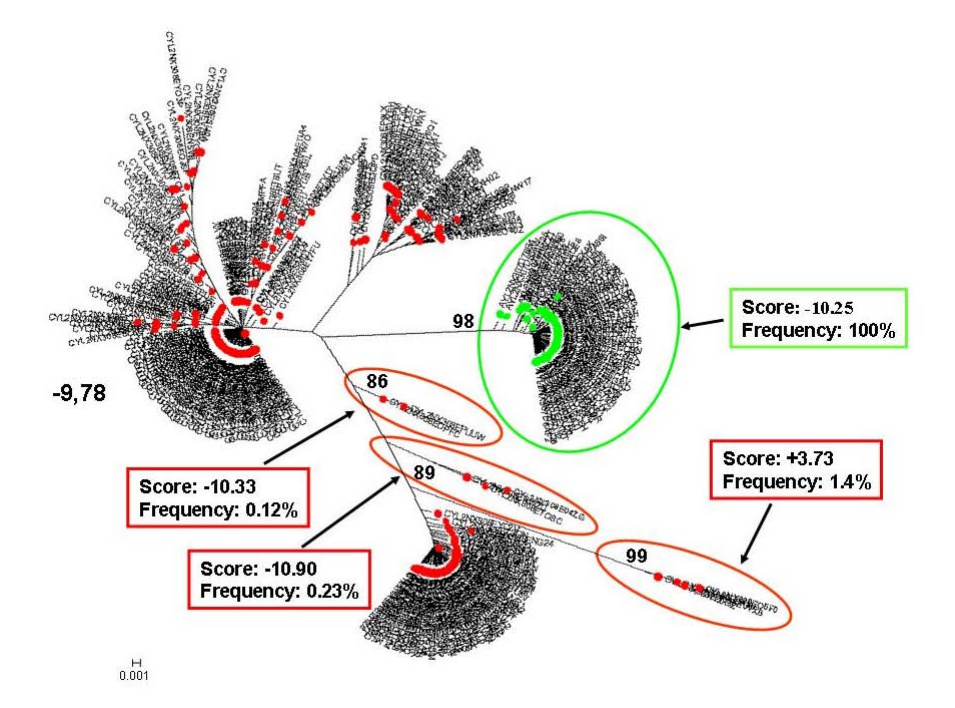
**Individual phylogenetic tree of HIV-1 V3 amino acid sequences from Pt.2**. Proviral quasispecies harboured by monocytes (red circle = CD36-provirus) and T lymphocytes (green circle = CD26-provirus) of Pt.2 were analyzed, as in Fig. 1.

**Figure 3:**
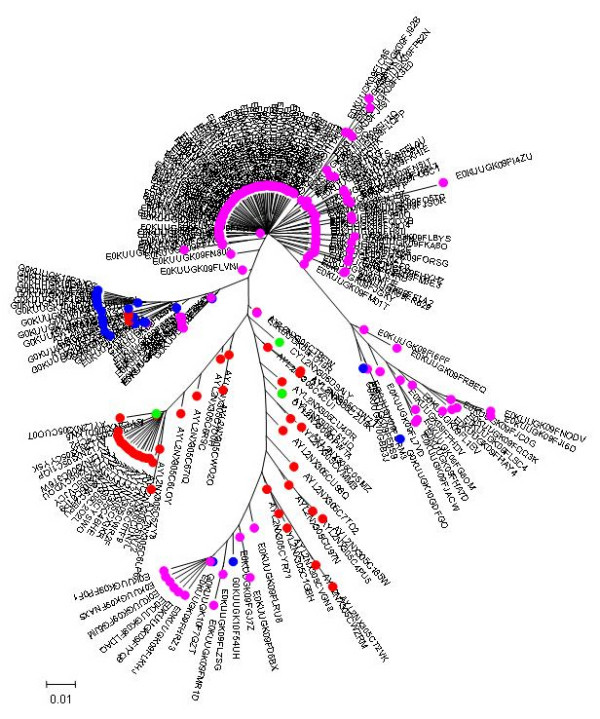
**Individual phylogenetic tree of HIV-1 V3 amino acid sequences from Pt.3**. Proviral quasispecies harboured by monocytes (red circle = CD36-provirus) and T lymphocytes (green circle = CD26-provirus) of Pt.3 were analyzed as in Fig. 1. In addition the analysis also included from the same patient virions rebounding into the circulation one month after therapy interruption and sorted by immunocapture with monoclonal antibodies to CD36 (blue circle = CD36-virus) and CD26 (purple circle = CD26-virus).

**Figure 4:**
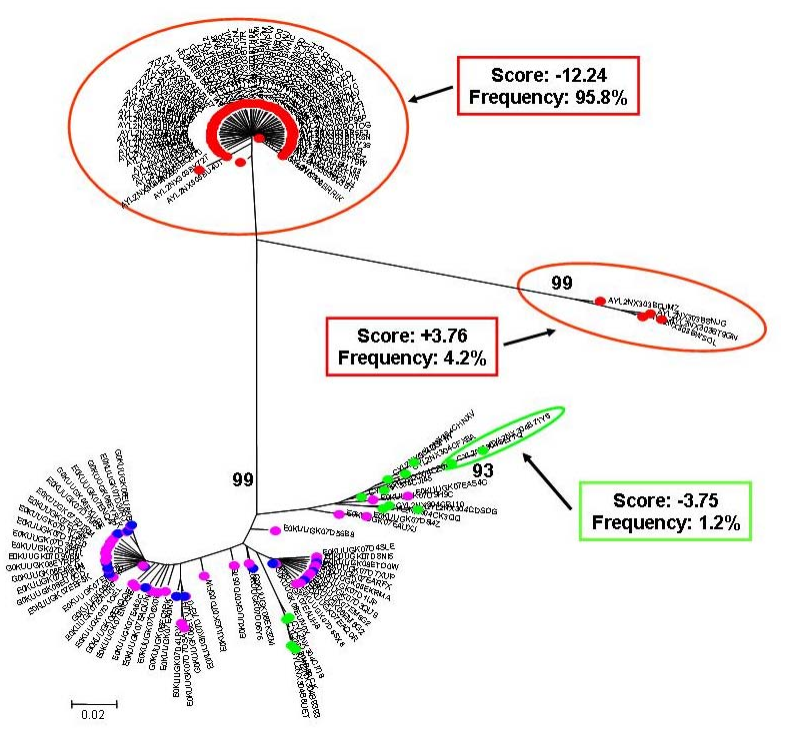
**Individual phylogenetic tree of HIV-1 V3 amino acid sequences from Pt.4**. Proviral quasispecies harboured by monocytes (red circle = CD36-provirus) and T lymphocytes (green circle = CD26-provirus), virions rebounding into the circulation one month after therapy interruption (blue circle = CD36-virus) and CD26 (purple circle = CD26-virus), from Pt.4 were analyzed as in Fig. 3.

**Figure 5:**
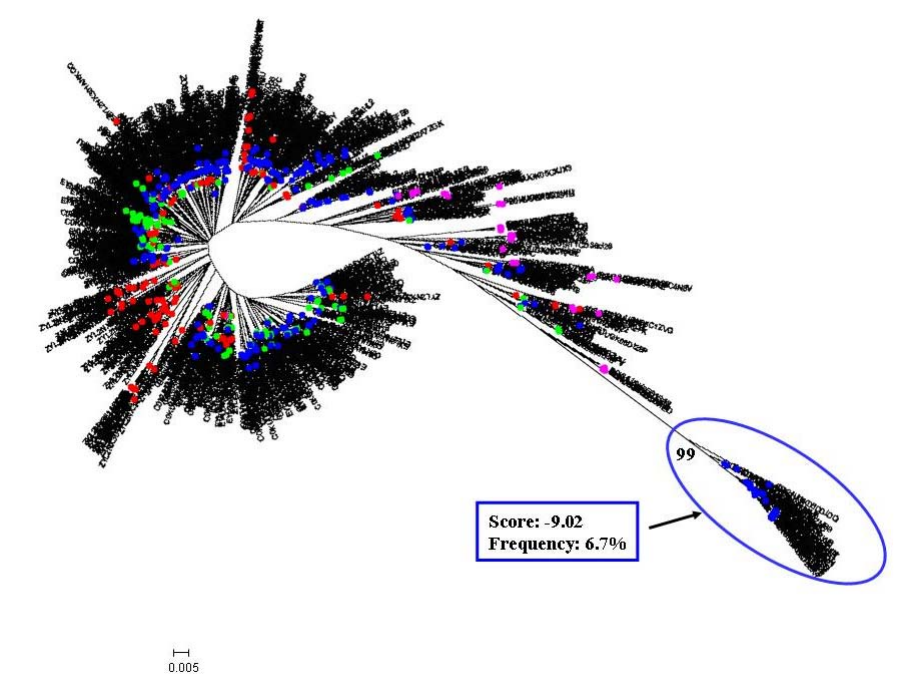
**Individual phylogenetic tree of HIV-1 V3 amino acid sequences from Pt.5**. Proviral quasispecies harboured by monocytes (red circle = CD36-provirus) and T lymphocytes (green circle = CD26-provirus), virions rebounding into the circulation one month after therapy interruption (blue circle = CD36-virus) and CD26 (purple circle = CD26-virus), from Pt.5 were analyzed as in Fig. 3.

The repertoire of V3 amino acid sequences from each patient was also used to build individual phylogenetic trees that are shown in Figures 1, 2, 3, 4 and 5.

On the whole, consistent with heterogeneity data, the phylogenetic trees showed a more heterogeneous quasispecies in provirus harboured by CD36 cells than in provirus harboured by CD26 cells. Both monocytes and T lymphocytes might contribute to virus rebounding in the circulation after one month from therapy interruptions, although other virus sources might be also involved. In 3 out of 5 patients (Fig. 1, 2 and 4) monophyletic clusters of sequences deriving from each of the cell source could be highlighted. On the basis of the phylogenetic relationships between virions shed into the circulation and the proviral sequences archived in the two cellular sources, 3 distinct situations could be identified in the 3 patients where this analysis could be performed: i) in Pt. 4 (Fig. 4) the circulating virus (carrying either CD26 or CD36 on its surface) segregated with provirus harboured by T lymphocytes, implying that, apparently, the provirus harboured by monocytes before therapy interruption did not contribute to viremia rebound, at least at this time point; ii) in Pt. 3 (Fig. 3) both archived and replicating virus sequences were interspersed, preventing the identification of reservoir-specific proviral clusters; iii) in Pt. 5 (Fig. 5), the situation was similar to Pt. 3, but in addition, a segregating cluster of circulating virions displaying the CD36 surface marker could be identified that seemed not to be related to the provirus harboured by either circulating cell compartment. It is possible that the lack of separation of proviral quasispecies from CD36 and CD26 cells in patients 3 and 5 simply reflects a mere artefact. However, in our opinion, the contamination of CD36 provirus with lymphocyte-derived viruses is unrealistic, since data from Table 2 suggest that CD36 provirus from these two patients had greater variability (as both n. of variants and diversity) than the corresponding CD26 provirus, thereby ruling out that the CD36 provirus was contaminated by CD26 provirus. The proportionally fewer proviral sequences in monocytes is a more likely explanation. In fact, in the present study as in a previous one from our group [31], proviral load in monocytes was about 1/5 of that present in T lymphocytes. However, also to this regard the higher variability of CD36 provirus renders unlikely that a mere disproportion between the proviral quasispecies from the two cell compartments may fully explain the lack of separation.

### Co-receptor usage prediction of viral quasispecies

**Figure 6:**
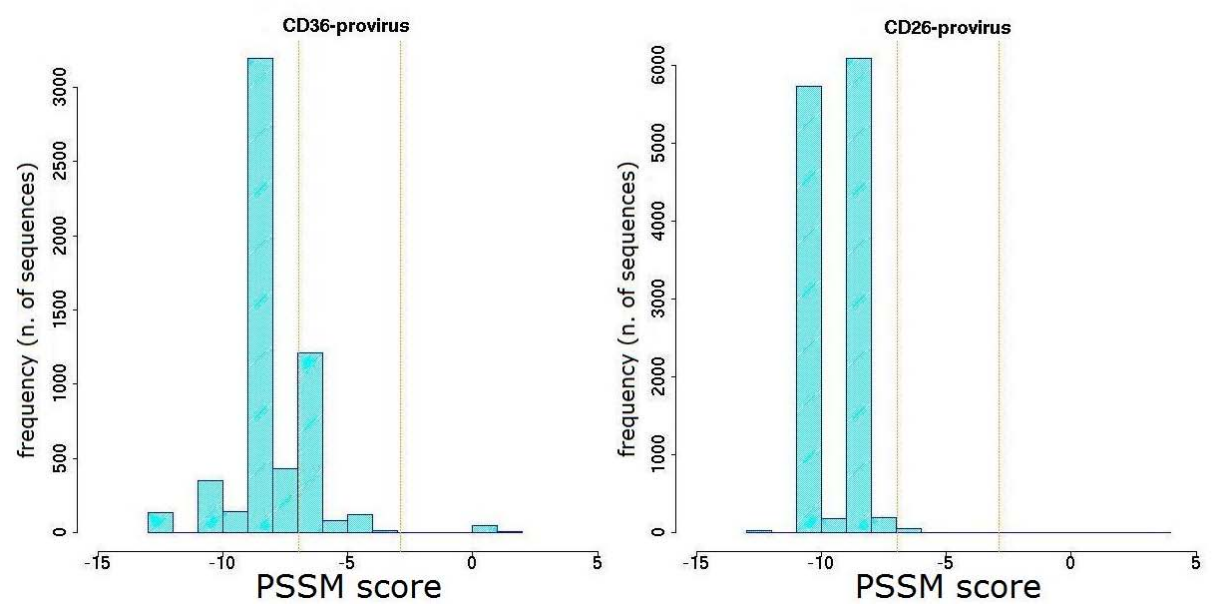
**PSSM score distribution of V3 amino acid sequences from Pt.1**. The PSSM score distribution of all the detected variants from CD36- and CD26-provirus present in Pt.1 at the moment of therapy interruption are shown. Vertical orange lines indicate 95th and 5th percentiles for CXCR4 and CCR5 predicted co-receptor usage (> -2.88 and < -6.96 respectively).

**Figure 7:**
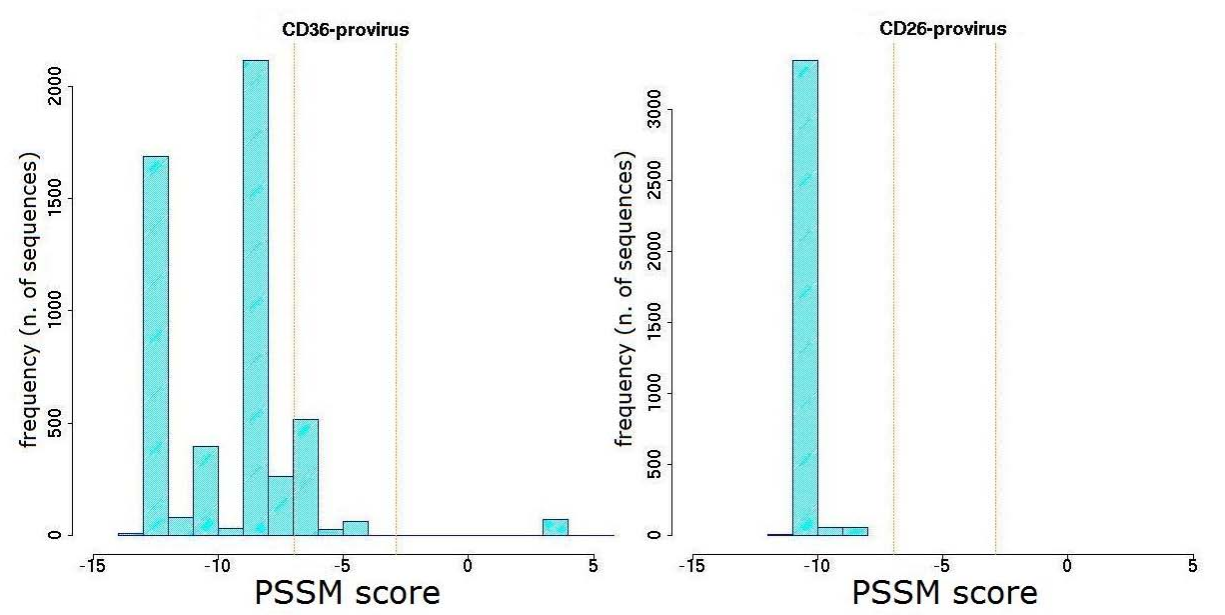
**PSSM score distribution of V3 amino acid sequences from Pt.2**. The PSSM score distributions of all the variants from CD36- and CD26- provirus detected in Pt.2 at the moment of therapy interruption are shown. Vertical orange lines indicate percentiles for predicted co-receptor usage as in Fig. 6.

**Figure 8:**
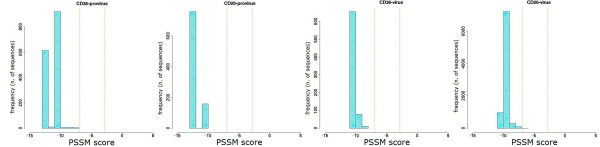
**PSSM score distribution of V3 amino acid sequences from Pt.3**. The PSSM score distribution of all variants from CD36- and CD26-provirus detected in Pt.3 at the moment of therapy interruption are shown together with those from CD36- and CD26- captured virus present one month after therapy stop. Vertical orange lines indicate percentiles for predicted co-receptor usage as in Fig. 6.

**Figure 9:**
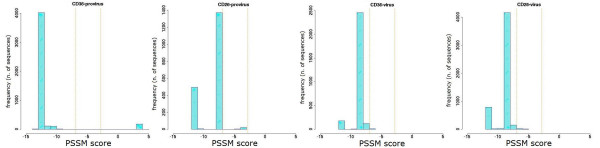
**PSSM score distribution of V3 amino acid sequences from Pt.4**. The PSSM score distribution of all variants from CD36- and CD26-provirus detected in Pt.4 at the moment of therapy interruption are shown together with those from CD36- and CD26-captured virus present one month after therapy stop. Vertical orange lines indicate percentiles for predicted co-receptor usage as in Fig. 6.

**Figure 10:**
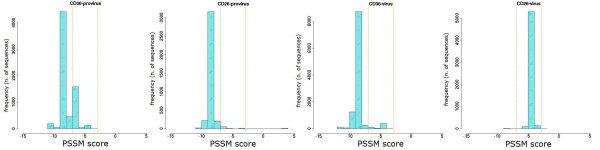
**PSSM score distribution of V3 amino acid sequences from Pt.5**. The PSSM score distribution of all variants from CD36- and CD26-provirus detected in Pt.5 at the moment of therapy interruption are shown together with those from CD36- and CD26-captured virus present one month after therapy stop. Vertical orange lines indicate percentiles for predicted co-receptor usage as in Fig. 6.

The sequence repertoire obtained by ultra-deep pyrosequencing was also used to obtain a prediction of co-receptor usage, by PSSM analysis of V3 loop amino acid sequences. The distribution of variants according to their score value for each patient is shown in Figures 6, 7, 8, 9 and 10. Some general findings could be identified. For both proviral and circulating viral genomes in monocytes and T lymphocytes, the results indicated a predominant CCR5 coreceptor usage (R5 phenotype). However, the intra-patient range of PSSM score values was rather wide, particularly in the archived proviral sequences, highlighting the presence of minority sequences (from <0.1% to 4.2%) with a clear CXCR4 coreceptor usage (X4 phenotype) only in archived sequences from at least one cellular reservoir in 4 patients (Fig 6, 7, 9, 10), while only one patient (Pt. 3, Fig. 8) harboured exclusively R5 populations. None of the patients showed X4 variants in circulating virus, as the minimum value obtained by PSSM prediction was -2.99.

In addition, we calculated the mean score for the sample-specific clusters segregating with bootstrap values ≥ 80% to see if the phylogenetic segregation could be associated with different biological properties, namely coreceptor usage. Individual scores of segregating clusters from each sample type are shown in the inserts of Fig. 1, 2, 3, 4, 5, together with their relative abundance in the corresponding sample type. It is noteworthy that two clusters of CD36-provirus, identified in Pt. 2 and Pt. 4 (Fig. 2 and 4), displayed PSSM scores clearly predicting X4 phenotype (+3.73 and +3.76). However, these clusters represented only 1.4% and 4.2%, respectively, of all CD36-provirus sequences in the corresponding patients. We also found a single cluster of CD26-provirus in Pt. 4, including only 1.2% of CD26-provirus sequences, with a mean PSSM score of -3.75, when mean CD26 provirus score was -8.40.

Concerning circulating virus, in Pt. 5 less negative score values were observed for CD26-virus (-4.85) as compared to CD36-virus (-8.43). In addition, in this patient the segregating cluster of CD36-virus presented a mean score value (-9.02) (Fig. 5) more negative than the mean values of CD36-virus. Also in this case, this cluster included only a minority (6.7%) of all CD36-virus sequences.

## Discussion

This study provided a direct comparison between the HIV-1 quasispecies archived in monocytes and T lymphocytes with that present in circulating virions replicating in the same cell types after one month from therapy interruption, exploiting the presence of cell lineage markers on viral envelope. In fact, this method proved to be highly specific in sorting the virions originating from monocytes and CD4 T lymphocytes, respectively. The amount of plasma virions captured by anti-CD26 and -CD36 antibodies ranged between 5 and 10%. However, a similar proportion of virions immuno-captured by these two antibodies did not necessarily mean that the amount of virions carrying these molecules was similar. In fact, a number of factors may explain this apparent paradox, including the asymmetric position of cell-derived molecules on the virion envelope, and the different affinity of the monoclonal antibodies used for the immuno-capture, as claimed in previous study [32].

The analysis took advantage of ultra-deep pyrosequencing based on the powerful technology of massively parallel sequencing that enabled us to perform a very detailed analysis of viral quasispecies. The data set obtained for each patient is some order of magnitude higher than any previously studied with conventional approaches based on cloning of PCR products or on limiting dilution PCR [11,13-15]. This allowed us to analyze previously inaccessible aspects of HIV-1 quasispecies, such as coreceptor usage of minority variants present in archived proviral sequences and in actually replicating virions.

One of the main findings of this study is that the heterogeneity of provirus associated with monocytes/macrophages after prolonged HAART is generally higher than that of T lymphocytes, even though in this study the proviral load in monocytes was about 1/5 of that present in T lymphocytes, in agreement with our previous findings [31].

It has been previously shown that during therapy, HIV-1 variants harboured by monocytes may evolve more rapidly than those harboured by T cells [11,14], eventually leading to segregation of viral quasispecies present in the two different cell types. This is probably due to the fact that monocytes and T lymphocytes may show different sensitivity to individual antiviral drugs, with residual ongoing viral replication in monocytes also in the presence of suppressive therapy. However, a greater diversity in HIV quasispecies harboured by monocyte/macrophages may be also due to other possibilities, including larger numbers of locally replicating quasispecies prior to treatment. In favour of this hypothesis recent data by Joos et al. [33] showed that rebounding virus after therapy interruption was phylogenetically older than virus present at the moment of therapy start. These findings argued against the possibility that rebounding virus during therapy interruptions originated from viral populations undergoing low-level persistent replication during suppressive therapy Instead, they are consistent with the hypothesis of virus reactivation from latent reservoirs.

R5 variants are generally predominant in our patients, even long after primary infection. This is in accordance with numerous studies showing that most of the patients' isolates, even at late stages of infection, are generally R5/monocytotropic or R5/X4 dual-tropic [34,35]. Furthermore, the results of the present study indicated that circulating monocytes and T lymphocytes may harbour minor provirus variants predictive of CXCR4 usage, as previously shown *ex vivo *for tissue resident macrophages or *in vitro *for monocytes-derived-macrophages [36,37]. The fact that the two patients displaying clusters of sequences predictive of CXCR4 usage in proviral HIV DNA in monocytes have been on fully suppressive HAART for almost the entire time of the infection, although intriguing, is not completely unexpected, as in a recent study where X4 viruses (either pure or dual/mixed R5/X4) were recognized in 17.2% of patients studied early after seroconversion [38].

It is not possible to deduce information on the replicative competence of archived provirus from these molecular data. In fact, one limitation of the next generation pyrosequencing technology is that the clonal sequences may not be physically separated from the whole quasispecies. Hence, it would not be possible to conduct phenotypic analysis of clones unless the synthetic approach was used to re-construct the viral genomes to undergo biological characterization.

The small number of patients under study and the variable profile of viral quasispecies impair the possibility to make general conclusions from the present results. Nevertheless, the higher HIV-1 heterogeneity in monocytes and the presence of distant variants suggests that this compartment, although quantitatively inferior to the CD4 T cell reservoir [31], may represent a possible source of viral diversity and contribute to escape mechanisms in regards to both immunity and antiviral therapy. This idea may be clinically relevant in the light of the recent therapeutic approaches involving co-receptor antagonists [39] that required a deep profiling of the co-receptor usage [40].

Moreover, we could obtain direct evidence that both T lymphocytes and monocytes do actually contribute to virus rebounding in the circulation early after therapy interruption. However, at the same time we found that minor replicating clusters may derive from cellular sources different from the main circulating reservoirs. This is in agreement with the results of a recent study where quasispecies analysis of residual viremia present in patients on HAART was established and compared to those found in resting T CD4 cells [41].

## Conclusion

The combined use of immuno-capture of circulating virions associated with the ultra-deep sequence analysis of V3-containing *env *region may be a powerful tool to investigate viral dynamics, useful for exploring the contribution of different viral reservoirs to replicating virus along the natural history of the infection, and for identifying co-receptor usage in minority viral populations harboured by different cell lineages.

## Competing interests

The authors declare that they have no competing interests.

## Authors' contributions

GR, IA, MRC designed the study, wrote and drafted the manuscript. GR and IA performed immuno-capture of the virus and molecular experiments. AB collected and assembled the data. MP and GC contributed to the analysis of the data. GD, CV, PN and GI were the clinical referents of the study. All authors read and approved the final manuscript.

## Supplementary Material

Additional file 1 **Figure S1 – Phylogenetic tree obtained from anti-CD36 and anti-CD26 immunocapture of an artificial mixture of R5 and X4 laboratory strains.** Two HIV-1 strains, genetically distinct and with different coreceptor usage (i.e. the reference R5 strain HIV-1 BaL and a clinical X4 isolate, whose V3 loop sequences are CTRPNNNTRKSIHIGPGRAFYTTGEIIGDIRQAHC, PSSM score: -12.35, and CTRPNNNTRRRMTAGPGRVYYTTGQIVGDIRKAHC, PSSM score: +3.68, respectively) were grown the first on monocytes derived macrophages (MDM) and the second on PHA activated CD4+ T lymphocytes. The R5 and the X4 viral preparations were mixed at a ratio of 1:9 and applied to 2 sets of immunocapture wells, coated with anti-CD36 and anti-CD26 antibodies, respectively; then the immunocaptured virions were analyzed by ultra-deep pyrosequencing. For comparison, the sequences obtained by ultra-deep sequencing of the R5 and X4 strains (before mixing) were included in the tree. The results indicated that >99% of the sequences captured by anti-CD36 and anti-CD26 clustered with the R5 and X4 sequences, respectively. On the contrary, only one sequence variant, representing 0.51% of virions captured by anti-CD36, segregated with the X4 sequences, and only one sequence, representing 0.12% of virions captured by anti-CD26, clustered with the R5 sequences, suggesting very low level of cross contamination. Symbols: red circle = virions captured by anti-CD36; green circle = virions captured by anti-CD26; yellow circle = R5 BaL strain; blue circle = X4 clinical isolate Click here for file

Additional file 2 **Figure S2 – Reference plasmid flowgram.** Graphical representation of the region sequenced by the Sanger method and by pyro-sequencing. The 5' and 3' termini discarded by the correction procedure described in the Materials and Methods section are shaded in grey. Coverage of the single nucleotides is shown with a cyan line. Homopolymeric regions are shaded with pink boxes and sequencing errors are indicated by histogram bars with the following colour code: T-red, G-black, C-blue, A-green, Del-grey. The sequence obtained by the Sanger sequencing is shown at the bottom. Click here for file

Additional file 3 **Table S1**. Total starting nucleotide reads, filtered amino acid sequences, obtained after the application of the correction algorithm described in Materials and Methods section, and number of total unique variants for each sample type. Click here for file
