# Supplementary figures and images for: Massively parallel pyrosequencing highlights minority variants in the HIV-1 env quasispecies deriving from lymphomonocyte sub-populations
Source: Retrovirology. 2009 Feb 12;6:15. doi: 10.1186/1742-4690-6-15 (PMC2660291; doi:10.1186/1742-4690-6-15)

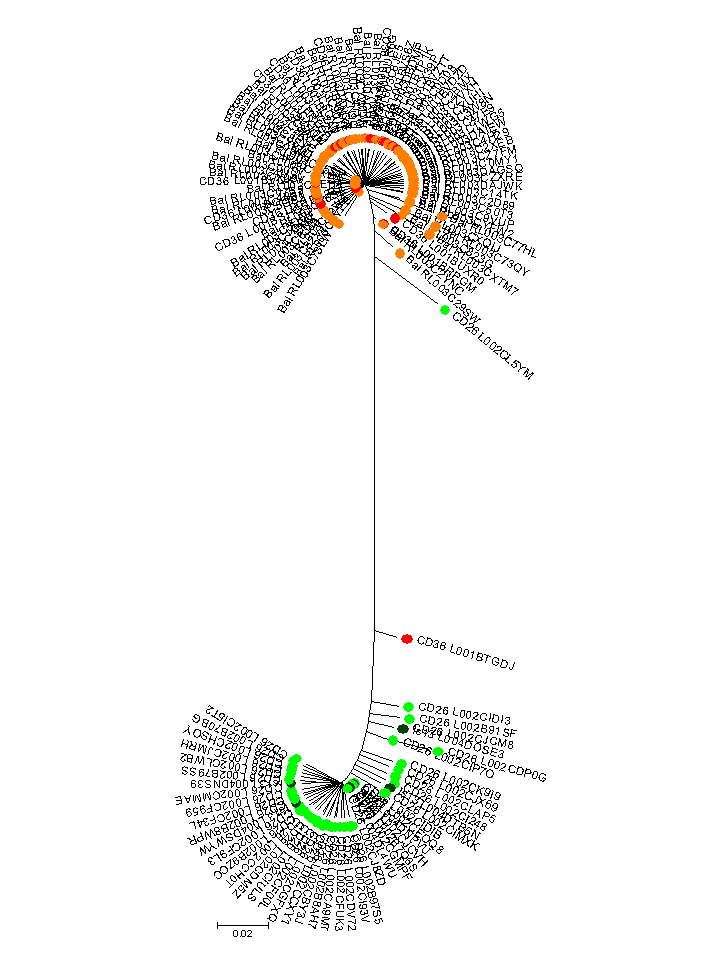

Supplement: Additional file 1 — Figure S1 – Phylogenetic tree obtained from anti-CD36 and anti-CD26 immunocapture of an artificial mixture of R5 and X4 laboratory strains. Two HIV-1 strains, genetically distinct and with different coreceptor usage (i.e. the reference R5 strain HIV-1 BaL and a clinical X4 isolate, whose V3 loop sequences are CTRPNNNTRKSIHIGPGRAFYTTGEIIGDIRQAHC, PSSM score: -12.35, and CTRPNNNTRRRMTAGPGRVYYTTGQIVGDIRKAHC, PSSM score: +3.68, respectively) were grown the first on monocytes derived macrophages (MDM) and the second on PHA activated CD4+ T lymphocytes. The R5 and the X4 viral preparations were mixed at a ratio of 1:9 and applied to 2 sets of immunocapture wells, coated with anti-CD36 and anti-CD26 antibodies, respectively; then the immunocaptured virions were analyzed by ultra-deep pyrosequencing. For comparison, the sequences obtained by ultra-deep sequencing of the R5 and X4 strains (before mixing) were included in the tree. The results indicated that >99% of the sequences captured by anti-CD36 and anti-CD26 clustered with the R5 and X4 sequences, respectively. On the contrary, only one sequence variant, representing 0.51% of virions captured by anti-CD36, segregated with the X4 sequences, and only one sequence, representing 0.12% of virions captured by anti-CD26, clustered with the R5 sequences, suggesting very low level of cross contamination. Symbols: red circle = virions captured by anti-CD36; green circle = virions captured by anti-CD26; yellow circle = R5 BaL strain; blue circle = X4 clinical isolate [file 1742-4690-6-15-S1.tiff]

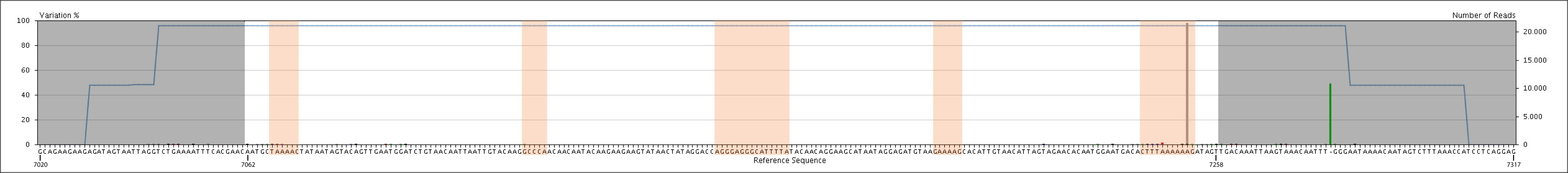

Supplement: Additional file 2 — Figure S2 – Reference plasmid flowgram. Graphical representation of the region sequenced by the Sanger method and by pyro-sequencing. The 5' and 3' termini discarded by the correction procedure described in the Materials and Methods section are shaded in grey. Coverage of the single nucleotides is shown with a cyan line. Homopolymeric regions are shaded with pink boxes and sequencing errors are indicated by histogram bars with the following colour code: T-red, G-black, C-blue, A-green, Del-grey. The sequence obtained by the Sanger sequencing is shown at the bottom. [file 1742-4690-6-15-S2.tiff]
