# Supplementary material for: Massively parallel pyrosequencing highlights minority variants in the HIV-1 env quasispecies deriving from lymphomonocyte sub-populations
Source: Retrovirology. 2009 Feb 12;6:15. doi: 10.1186/1742-4690-6-15 (PMC2660291; doi:10.1186/1742-4690-6-15)
Supplement: Additional file 3 — Table S1. Total starting nucleotide reads, filtered amino acid sequences, obtained after the application of the correction algorithm described in Materials and Methods section, and number of total unique variants for each sample type. [file 1742-4690-6-15-S3.doc]

**Supplementary data**

**Supplementary Table 1. Total starting nucleotide reads, filtered amino acid sequences, obtained after the application of the correction algorithm described in Materials and Methods section, and number of total unique variants for each sample type.**

| **Patient** | **Sample type** | | **N. of starting nucleotide reads** | **N. of filtered amino acid sequences** | **N. of unique amino acid variants** |
| --- | --- | --- | --- | --- | --- |
| Pt.1 | Provirus | CD36 | 10,158 | 6,799 | 161 |
|  |  | CD26 | 17,650 | 13,044 | 226 |
| Pt.2 | Provirus | CD36 | 13,753 | 7,015 | 231 |
|  |  | CD26 | 12,646 | 3,991 | 59 |
| Pt.3 | Provirus | CD36 | 4,991 | 2,130 | 46 |
|  |  | CD26 | 13,866 | 1,599 | 3 |
|  | Virus | CD36 | 10,796 | 1,163 | 26 |
|  |  | CD26 | 16,237 | 9,866 | 158 |
| Pt.4 | Provirus | CD36 | 7,638 | 5,045 | 76 |
|  |  | CD26 | 12,486 | 3,262 | 14 |
|  | Virus | CD36 | 9,445 | 3,546 | 15 |
|  |  | CD26 | 16,875 | 6,547 | 41 |
| Pt.5 | Provirus | CD36 | 12,645 | 7,543 | 157 |
|  |  | CD26 | 10,271 | 4,625 | 120 |
|  | Virus | CD36 | 19,059 | 12,504 | 305 |
|  |  | CD26 | 20,643 | 6,668 | 38 |
